# Fluorescent carbon dots from mono- and polysaccharides: synthesis, properties and applications

**DOI:** 10.3762/bjoc.13.67

**Published:** 2017-04-10

**Authors:** Stephen Hill, M Carmen Galan

**Affiliations:** 1 School of Chemistry, University of Bristol, Cantock’s Close, Bristol BS8 1TS, UK

**Keywords:** fluorescent carbon dots, monosaccharides, nanomaterials, nanotechnology applications, polysaccharides

## Abstract

Fluorescent carbon dots (FCDs) are an emerging class of nanomaterials made from carbon sources that have been hailed as potential non-toxic replacements to traditional semiconductor quantum dots (QDs). Particularly in the areas of live imaging and drug delivery, due to their water solubility, low toxicity and photo- and chemical stability. Carbohydrates are readily available chiral biomolecules in nature which offer an attractive and cheap starting material from which to synthesise FCDs with distinct features and interesting applications. This mini-review article will cover the progress in the development of FCDs prepared from carbohydrate sources with an emphasis on their synthesis, functionalization and technical applications, including discussions on current challenges.

## Introduction

Nanotechnology applied to biological and biomedical problems has seen an explosion of research in recent years [1]. Functional nanomaterials that can carry biologically relevant molecules have become very useful for drug delivery, sensing and catalysis to name just a few applications. As a result, nanomaterials exhibiting novel electronic and optical properties, having controlled size, geometry, surface distribution and functionality have been developed as materials for probing biological interactions and in biomedical applications [2–6]. Among these novel type of probes, luminescent semiconductors, quantum dots (QDs), which possess a narrow emission spectra and common excitation, superior photostability and electron density when compared to organic fluorophores, in addition to bright visible emission, have become particularly popular for their versatility as non-isotopic detection labels which are amenable to live cell imaging and immunoassay applications [7]. In particular, cadmium-based QDs (e.g., CdS, CdSe, CdSe/ZnS) are commonly used for in vitro biological studies due to their well-established synthesis and functionalisation strategies, tuneable emission profiles and high quantum yields of fluorescence (QYs) [8–11]. However, the presence of heavy metals like Cd^2+^, and the associated concerns surrounding heavy metal toxicity has meant that their in vivo applications are restricted [12]. Therefore, the development of fluorescent nanoparticles that are able to replicate QD fluorescence properties without exhibiting long term toxicity profiles, has become very relevant.

The term carbon dots (CDs) has been coined to describe a new class of carbon-based nanomaterials which are typically discrete, quasi-spherical nanoparticles, with sizes usually less than 10 nm in diameter (although bigger sizes have recently been reported). These relatively new nanomaterials have found many applications in the fields of photo- and electrocatalysis, chemical sensing, biosensing, bioimaging and nanomedicine, due to their unique tuneable photoluminescence (PL) properties, chemical inertness, high water solubility, ease and low cost fabrication and more importantly, low toxicity profiles. The latter makes these fluorescent nanomaterials attractive for a wide range of in vivo applications, which has been the topic of several recent reviews [13–15]. Following the serendipitous discovery by Xu et al. during the separation and purification of single-walled carbon nanotubes (SWCNTs) [16], the development of synthetic methodologies to access these fluorescent nanomaterials combined with their myriad of applications, has led to CDs being hailed as the potential non-toxic successors to traditional semiconductor QDs, particularly in the areas of live imaging and drug delivery.

Synthetic approaches to access CDs can be classified into two broad categories: top-down or bottom-up syntheses. Top-down methods are characterised by using a bulk carbon substrate as the starting material; using conditions that remove nanoparticles from the bulk substrate such as electrochemistry, chemical oxidation, arc discharge or laser ablation, carbon-based nanoparticles can be obtained. Typical substrates used are single/multi-walled carbon nanotubes, graphite, graphene or candle soot, amongst many others [15,17]. The crystalline make-up of top-down derived CDs is usually highly sp^2^ in character, which is transferred from the sp^2^-enriched starting materials, e.g., graphite or graphene. Conversely bottom-up methodologies rely on the use of a molecular precursor which can be treated in such a way as to seed the formation of a CD. Typical starting materials include amino acids, citric acid, biomass and carbohydrates to name but a few, which can be reacted using thermal decomposition, chemical or hydrothermal oxidation, microwave, acid-mediated reflux, ultrasonic irradiation or silica nanoparticle-templated synthesis [18–23]. Unlike their top-down equivalents, the CDs derived from these methods are usually less sp^2^ crystalline and tend to have more amorphous morphologies. It should be stated that no two CD preparations lead to the same type of nanoparticle, as any changes to the ratio and composition of starting materials, additives, solvent, temperature, type of vessel, etc., does have an effect on the final molecular composition and architecture of the CD. Resultantly, differential properties are easily acquired through minor manipulations of the CD synthesis. To date, the de novo rational design of bottom-up syntheses of CDs for advanced application is limited in the literature.

Carbohydrates are one of the most diverse and important class of biomolecules in nature and offer well-defined chiral scaffolds primed for modification at the anomeric position and alcohol functionalities. Therefore, the use of carbohydrates as a starting material for synthesizing CDs is extremely attractive not only due to their abundance, availability and heterogeneity, but also due to their high water solubility, low-carbonisation temperatures, low cost and typically inherently lack toxicity. With all these options available to tune the synthesis of CDs, it is no surprise that researchers have already began to see the benefits of carbohydrates when considering the synthesis of novel FCDs with improved properties. For example, simple monosaccharides such as glucose, glucosamine, mannose, fructose and their derivatives and common disaccharides, e.g., sucrose, lactose, and maltose have been employed to prepare fluorescent carbon dots (FCDs) using different methodologies [13,24]. Similarly, important carbohydrate-based biopolymers such as cellulose, chitin, chitosan, dextran, cyclodextrin, and hyaluronic acid, which differ not only in elemental composition, but also in chemo-physical properties, have also been successfully utilised in the preparation of CDs, where their differences allow tailoring of the CD structure and properties [25].

In this review, we focus on the most recent approaches developed to prepare fluorescent CDs using mono-, oligo- and polysaccharides as the main carbon source.

## Review

### Fluorescent carbon dots synthesised from monosaccharides

#### Glucose-based fluorescent carbon dots

Sustainable syntheses of CDs have driven researchers to find readily available, cheap and renewable carbon sources of which the monosaccharide glucose is an ideal candidate. Not only is glucose cheap and commercially available, but also has a low carbonisation temperature, ring-opens readily to afford a reactive aldehyde moiety which can be further exploited for conjugations, polymerisations and (hetero)aromatic formation, which are all ideal for generating CDs [26–27]. For these reasons, in addition to the inherent low toxicity and high water solubility of glucose, this particular monosaccharide has been extensively used as an ideal carbon source for CD formation, under a range of experimental conditions.

**Scheme 1:**
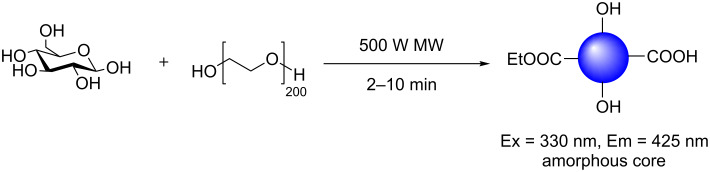
Microwave-driven reaction of glucose in the presence of PEG-200 to afford blue-emissive CDs.

The microwave-assisted synthesis of FCDs from a glucose solution in the presence of poly(ethylene glycol)-200 (PEG-200) by Yang et al. is, to the best of our knowledge, the first reported example involving a carbohydrate moiety (Scheme 1) [18]. The water-soluble nanoparticles exhibited an amorphous core, as deduced by X-ray diffraction (XRD), while Fourier-transformed infrared (FTIR) spectroscopy analysis indicated the presence of a range of oxygen-containing functionalities, e.g., alcohols, ethers and carboxylic acids on the CD surface, which are likely the reason for the high water solubility exhibited by the nanomaterial. This type of chemical profile is typical of standard bottom-up synthesised CDs [15,28]. Interestingly, the team was also able to show that the use of PEG-200, as a surface passivation agent (SPA), was crucial for favourable photoluminescence (PL) properties and QYs of up to 6.3% were achieved. The use of SPAs is among one of two main techniques that are widely employed to improve the PL properties of FCDs. SPAs are argued to provide uniform PL trapping sites on the CD surface, alongside promoting new functionality that can work, in tandem with the core, to turn-on fluorescence.

**Scheme 2:**
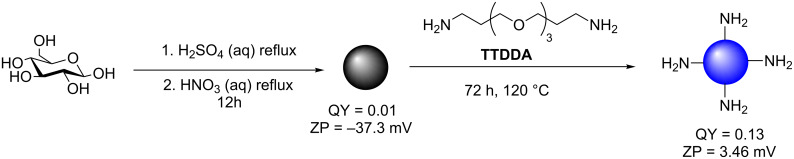
Two-step synthesis of TTDDA-coated CDs generated from acid-refluxed glucose.

Another example, which highlights the importance of surface passivation and how SPAs can be used to modify and tune CD chemical and physical properties, was reported by Travas-Sejdic et al. [23]. They also employed glucose as the carbon precursor which, after refluxing in aqueous H_2_SO_4_, yielded carbonaceous nanoparticles with observable PL (Scheme 2). Further treatment with aqueous HNO_3_ under reflux, yielded nanoparticles of weak PL (QY = 1%). The PL properties could be improved upon introducing surface passivation, which was achieved by heating the weakly fluorescent CDs in a solution of 4,7,10-trioxa-1,13-tridecanediamine (TTDDA) for 72 hours at 120 °C to give a nanomaterial with QY values of up to 13%. FTIR studies suggested that TTDDA incorporation onto CDs occurred via amide formation, from the reaction between surface carboxylic acids and the corresponding amine SPA. This was further supported by the change in zeta-potential (ZP) values which shifted from −37.3 mV (non-passivated CD) to 3.46 mV (TTDDA passivated CD). Similar carbonaceous materials were obtained when the team used sucrose or starch as starting carbohydrate materials.

**Scheme 3:**
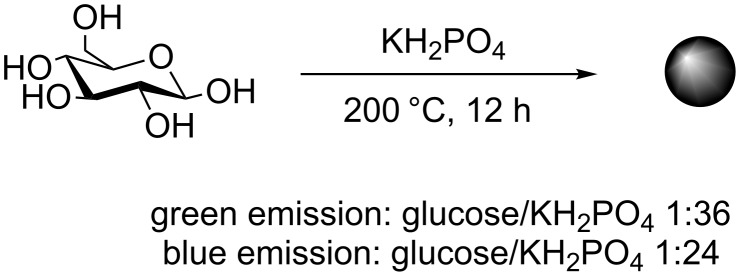
Glucose-derived CDs using KH_2_PO_4_ as a dehydrating agent to both form and tune CD’s properties.

In addition to microwave and acid reflux-mediated glucose dehydration reactions, the group of Wang developed an alternative protocol that combined glucose with monopotassium phosphate (KH_2_PO_4_) in a Teflon-lined autoclave chamber with heating to 200 °C for 12 h (Scheme 3) [29]. The fluorescence emission could be tuned by changing the ratio of sugar and KH_2_PO_4_. For instance a molar ratio of 1:26 (glucose/KH_2_PO_4_) afforded blue-fluorescent CDs (QY = 0.02), whereas a 1:36 ratio yielded green-fluorescent CDs (QY = 0.01). In the absence of KH_2_PO_4_, irregular black carbon aggregates were obtained. Raman and TEM analysis showed both types of FCDs had graphitic crystallinity. This example highlights that an inorganic-based dehydrating agent could be used instead of a traditional diamine SPA to induce CD dehydration and affect their PL properties. Most carbohydrate-derived CDs emit in the blue area of the visible section of the electromagnetic spectrum under UV/high energy blue excitation. However, most mammalian cells are also autofluorescent in this particular region [30]. As a result, the majority of CDs produced with blue emission have QYs that are not suitable for bioimaging applications. CDs with multicolour/excitation-dependent emission that can be red shifted and avoid the cellular autofluorescence window, are a good alternative. Unfortunately, the CD fluorescence tends to lose intensity upon red-shifting the excitation. An ideal CD probe for bioimaging applications will have either a high QY in the blue, or adequate green to red emission. Thus, the green-emissive glucose-based CDs produced by Wang et al. are ideal for this type of application and the team showed their applicability in cell internalisation studies with HepG2 cells [29]. The green CDs were non-toxic to cells at concentrations of up to 625 μg/mL and exposures of 72 h. Laser scanning confocal microscopy (LSCM) demonstrated cell internalization, making these materials a good candidate as a bioimaging agent.

In 2011 Qu et al. developed a tuneable synthesis of FCDs by selecting a different inorganic ion and carbohydrate combinations using microwave irradiation as the heating source, demonstrating that both the starting material and dehydrating agent of choice can allow tuning/manipulation of the fluorescence properties of the system [31]. It was found that irradiation times of 14 min could be employed to afford CDs from glycerol, glycol, glucose or sucrose. The source of the inorganic ion was important too, as increasing the valency of either the anion or cation would lead to a greater ability to dehydrate the carbon precursor. An ideal balance of cation and anion valence was found when using CuSO_4_ which afforded CDs with QY of up to 9.5%, in-line with the state-of-the-art at the time.

**Scheme 4:**
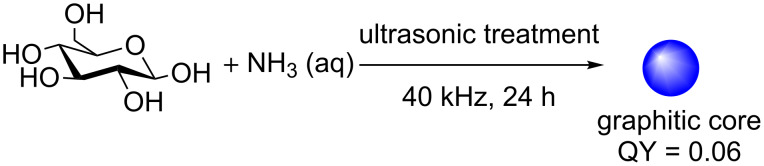
Ultrasonic-mediated synthesis of glucose-derived CDs in the presence of ammonia.

As an alternative, Kang et al. showed the following year that nitrogen-doped water-soluble fluorescent CDs could be afforded in a one-step ultrasonic reaction of glucose and aqueous ammonia [NH_3_ (aq)] in solution (Scheme 4) [32]. The CDs are generated by the formation of small vacuum bubbles in solution by alternating high and low pressure waves. This process leads to temperature increases, hydrodynamic shear forces and the formation of high-speed liquid jets in solution. All of these effects facilitate the degradation of glucose and the incorporation of ammonia into the CD structure. The introduction of N-doping allows the injection of electrons into the CD structure, which allows for new PL and fluorescence properties to be established. This is a widely employed strategy for improving the QY of CDs. The team was able to show that the presence of the dopant yielded N-doped CDs with a QY of 6%, which was superior to the N-free CDs. The resultant CDs afforded from the ultrasonic treatment were well dispersed, with TEM indicating graphite crystallinity with blue-green emission.

Most reported CD syntheses, regardless of the type of starting material or synthetic method, tend to produce CDs with blue-green fluorescence emission. Jana et al. reported a carbohydrate-based preparation to access yellow and red emissive CDs, demonstrating that fine-tuning the reaction conditions, combined with the use of additives, can lead to modifications in the emissive properties of the nanoparticles [20]. The team was able to show that the use of sulfuric acid with a carbohydrate, although the exact carbohydrate used is not disclosed in the article, generally gave blue-green emission, whereas phosphoric acid-mediated CD formation gave particles with red-shifted emission profiles (Red-CD). Interestingly, Red-CDs only had stable red emission in strong acidic conditions; changing to green emission in neutral or basic pH. The origin of the tuneable fluorescence was attributed, by the authors, to a number of contributing factors including the different sizes of the nanoparticles as key. As the CD size increases, the size of sp^2^ domains can also increase (controlled by the dehydrating agent). Additionally, the introduction of certain heteroatoms can allow for red-shifted emission with increasing size. Moreover, the presence of defect sites, which are associated to the PL properties, is confirmed using room temperature electron paramagnetic resonance (EPR) spectroscopy, by the presence of free electrons in the spectra. Further functionalization of the hydrophobic red fluorescent CDs via surface passivation and polymer coating, in which hydrophilic anhydride groups of the polymer can react with PEG-diamine, via a ring-opening to afford a free acid and an amide-linked SPA, lead to water-soluble CDs ready for bio-labelling applications. The CDs were then labelled with either TAT (a cell-penetrating peptide), or folate and then incubated with HeLa cells. Fluorescence microscopy images confirmed that incubation times of 3–6 hours were adequate to allow for CD labelling of the cells. Further toxicity assays indicated that concentrations of up to 200 μg/mL were tolerated, as determined by cell viability studies (MTT assay). More recently, the same team developed hydrophobic yellow and red emissive CDs via the degradation of ascorbic acid in the presence of oleophilic oleylamine [13]. The CDs were similarly polymer coated with poly(maleic anhydride-*alt*-1-octadecene) that could subsequently be functionalised with hydrophilic PEG-diamine, providing an amine functionality for conjugation with glucosamine, histidine, arginine and folate. The yellow/red emissive loaded-CDs were shown to be viable bioimaging probes in live cells, since their emission did not overlap with the cell autofluorescence.

**Scheme 5:**
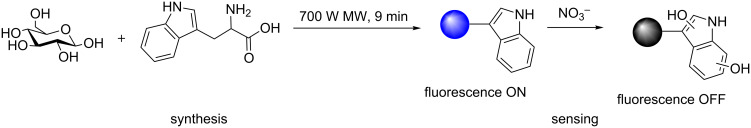
Tryptophan-derived CDs used for the sensing of peroxynitrite in serum-fortified cell media.

Recent years have seen an increase in synthetic reports of large scale N-doped CDs with good QYs from carbohydrate starting materials. For example in 2014, Leitão et al. described the microwave synthesis of CDs using 2.5 g of glucose and 0.3 g of tryptophan as the N-dopant/surface passivation agent (Scheme 5) [33]. The resultant CDs had a QY of up to 12% (34-times higher than that of the undoped CDs). Interestingly, the N-doped CDs in this report had a 20 nm diameter, as determined by TEM, which is contrary to the generally held belief that CDs’ particular properties are only observed below a diameter of 10 nm, which is not the case here and has since been observed in one other carbohydrate-derived CD synthesis [34]. The team demonstrated the utility of the glucose/tryptophan-derived CDs as a sensor of peroxynitrite anions (NO_3_^−^) in solution. The peroxynitrite anion is one of the key reactive species which is implicated in various metabolic and physiological processes [35]. Thus, it is important to provide analytical methods to detect and quantify its presence, however, due to its high reactivity, low concentration levels and quick diffusion, it has been traditionally difficult to detect. The team was able to show significant quenching of the CDs via tryptophan oxidation of the exposed residues on the surface of CDs (Scheme 5). Post-oxidation fluorescence is compromised and therefore can be used as a signal for selectively sensing peroxynitrite up to concentrations of 1.5 μM (with a linear regression between 2.5–50 μM). The sensing ability of the nanoparticles was exhibited in serum-fortified samples, which can be regarded as a biomimetic for complex biological media.

**Scheme 6:**
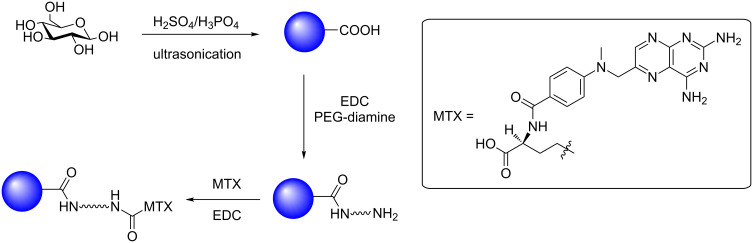
Glucose-derived CDs conjugated with methotrexate for the treatment of H157 lung cancer cells.

A number of glucose-based CDs has been reported in recent years as drug-delivery vehicles. In 2015, Yunus et al. synthesized CDs by the ultrasonication of glucose or sucrose in the presence of oxidising conditions afforded by H_3_PO_4_/H_2_SO_4_ (Scheme 6) [36]. The resultant CDs were blue emissive and the use of strong oxidising conditions during their synthesis afforded CDs with surface carboxylic acids that could be functionalised. Surface conjugation with PEG-diamine afforded a steric blocking, enhanced permeation and retention (EPR) shell, whilst providing an amine functionality for further surface conjugation. The anticancer drug methotrexate (MTX), which is a well-studied drug used to treat various types of cancer including lung cancer, was then conjugated via EDC-mediated amide coupling chemistry (Scheme 6) [37]. The MTX-CDs were internalised into H157 lung cancer cells and compared with cells exposed to unfunctionalised amine-bearing CDs. While the amine-CDs showed no cellular toxicity, MTX-CDs were highly toxic to H157 cell cultures, highlighting the potential applicability of carbohydrate-derived CDs as vehicles for the delivery of conventional cancer therapeutics.

**Scheme 7:**
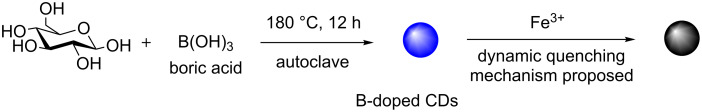
Boron-doped blue-emissive CDs used for sensing of Fe^3+^ ion in solution.

More recently, in addition to the introduction of electron-donating heteroatoms such as N or S as dopant agents to improve the PL properties of CDs, the use of boron as an additive, which is an electron-accepting element, has also been explored by Hao et al. [38]. The CDs were produced by the addition of boric acid (B(OH)_3_) into the hydrothermal carbonisation of glucose, using a Teflon autoclave at 180 °C for 12 h (Scheme 7). The resultant fluorescent nanoparticles had an average diameter of 4 nm and were negatively charged with ZP values of −40.7 mV. XPS and FTIR analysis confirmed the presence of B in the CD structure. Although, the addition of boron did not change the typical blue fluorescence profile significantly, when compared to other reported heteroatom-doping syntheses, the fluorescence of the B-CDs was dynamically quenched by Fe^3+^ ions. Mechanistic studies suggested that a dynamic quenching model was prevalent at low concentrations due to interactions between Fe^3+^ and the CD surface, possibly indicating the interception of an excited CD state by the Fe^3+^ ion that leads to fluorescence quenching. The group exemplified the applicability of the material by demonstrating the ability of the B-CD to sense Fe^3+^ in tap water samples with a limit of detection of 242 nM, which complies with U.S. Environmental Protection Agency standards.

**Scheme 8:**
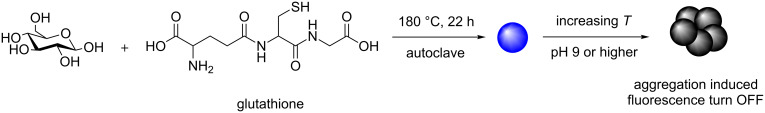
N/S-doped CDs with aggregation-induced fluorescence turn-off to temperature and pH stimuli.

Having shown that chemical doping with heteroatoms within the CD synthesis can lead to materials with improved PL and physicochemical properties, this has encouraged research groups to focus their efforts to study the effect of using different heteroatoms simultaneously. For example, in 2015 Zhang et al. demonstrated that the energy intensive, hydrothermal treatment of glucose in the presence of glutathione (acting as both SPA and N/S heteroatom dopant) conducted at 180 °C for 22 h, resulted in CDs with QYs of up to 7%. The obtained CDs had blue-emissive fluorescence under UV excitation, a standard feature of bottom-up produced carbohydrate-derived CDs (Scheme 8) [39]. Whereas most synthetic CDs from glucose sources generally show a fluorescence-decay response to one or several transition metals. Surprisingly, the CDs produced in the presence of glutathione had a very stable fluorescence output, which was unaffected by a wide-range of transition metal cations. The new CD’s fluorescence intensity was, however, sensitive to changes in both pH and temperature. The CDs were shown to aggregate and change emission from pH 3 to 9, which the authors attribute to the ionisation of the surface functionality (Scheme 8). The feature is reversible as demonstrated by monitoring the PL intensity at a given excitation at different pHs and during several iterations. Similarly the CDs were shown to have an emission-intensity dependence on the temperature. Upon increasing the temperature from 15 to 90 °C, 52% of the fluorescence was lost, without any red or blue shift in the emission maximum. The mechanism for this change was also attributed to nanoparticle aggregation, with CD agglomeration occurring at higher temperatures.

**Scheme 9:**
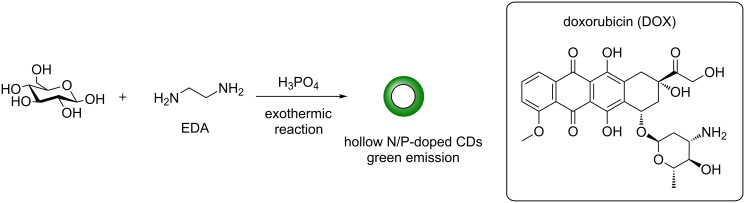
N/P-doped hollow CDs for efficient drug delivery of doxorubicin.

Two different groups reported, nearly concurrently their results, in the use of both N and P as dopants in their CD syntheses. For instance, Dong et al. described the development of dual-doped hollow CDs using a wet-chemical method from glucose in the presence of 1,2-ethylenediamine (EDA) and conc. H_3_PO_4_ (Scheme 9) [40]. During the exothermic reaction, a foam was produced which resulted in green-emissive N,P-doped CDs that were hollow and had a diameter of 10 nm as determined by HRTEM and AFM. Despite the hollowness of the CDs, the nanoparticles exhibit an excitation-dependent emission profile akin to other glucose-derived, bottom-up synthesised CDs. The material’s unique properties deemed them ideal candidates for drug-delivery purposes. The team chose doxorubicin (DOX) as the model drug for this purpose and loading of DOX onto the CD was demonstrated via a change in the ZP from −9.3 mV to −0.13 mV, suggesting that an electrostatic interaction between the positively charged amino group in DOX and the negatively charged groups on the CD surface can take place. Also van der Waals and π–π stacking interactions were attributed as contributing factors to the DOX loading and DOX incorporation into the hollow CD cavity. Drug release at acidic pHs, further supported the proposed electrostatic interactions between DOX and the CDs. Further studies showed initial efficacy of the DOX–CD adduct as a beneficial drug-delivery system, even in animal models.

**Scheme 10:**
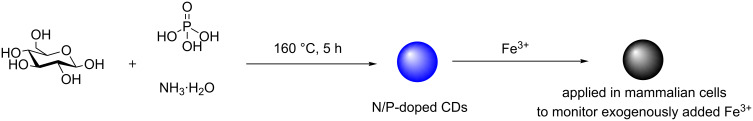
N/P-doped CDs applied to the sensing of Fe^3+^ ions in mammalian T24 cells.

On the other hand, Zhao et al. described an alternative synthesis for N/P-doped CDs. Hydrothermal oxidation of glucose, phosphoric acid and aqueous ammonia, as the nitrogen source, in a Teflon-lined autoclave followed by heating at 160 °C for 5 h afforded blue-emissive CDs under 365 nm excitation (Scheme 10) [41]. A high QY of 30% was obtained, which is one of the highest reported for a carbohydrate-derived CD to date. Interestingly, it was observed that the fluorescence of these N/P-CDs was strongly dependent on the local concentration of Fe^3+^. With increasing concentrations of the metal leading to fluorescence decay of the CDs, which was attributed to the interception of an excited state on the CD by the Fe^3+^ ion. The selectivity towards Fe^3+^ was demonstrated against a panel of other transition and alkali metals and a detection limit for Fe^3+^ of 1.8 nM was established. The glucose-derived blue-emissive CD could be readily internalised into T24 cells, without significant cell death, and used to detect the presence of exogenously added Fe^3+^ (Scheme 10).

It is important to highlight that small changes in the nitrogen source (EDA vs ammonia), ratio of reagents and reaction conditions can lead to marked differences in fluorescence, physical and chemical properties of the nanomaterials, as demonstrated with these two parallel reports for hollow-green and solid blue-emitting CDs.

**Scheme 11:**
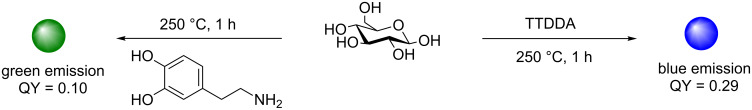
Comparative study of CDs formed from glucose and N-doped with TTDDA and dopamine.

A systematic study has been performed by the Travas-Sejdic group on the synthesis of CDs from either citric acid or glucose starting materials in the presence of either TTDDA or dopamine, in order to evaluate how the choice of carbon and nitrogen sources plays a key role in the final properties of these nanomaterials [42]. The authors found that the average size of CDs prepared was dependent on both, the carbon source, e.g., CDs from citric acid were larger than the ones derived from glucose, and the nitrogen source, e.g., CDs derived from dopamine were larger than those using TTDDA. The authors attribute this observation to the fact that citric acid possesses readily available carbonyl groups (as opposed to the masked aldehyde in the carbohydrate) that can readily react with basic TTDDA or dopamine to form stable intermediates; while glucose mostly interacts with the amine dopants through intramolecular forces such as van der Waals’ forces and hydrogen bonds. The latter weaker interactions cause the intermediates to break down into small fragments during the heating process resulting in smaller CDs. In the case of N-dopant agents, the presence of a bulky phenyl ring in dopamine was reasoned to be the possible cause for the somewhat larger sizes observed. In addition, it was found that PL properties were mostly dependent on the N-source, with optimum QY of up to 29.5% (for glucose) or 33.9% (for citric acid) when using TTDDA, as opposed to dopamine (Scheme 11). These results show that by experimentally probing the reaction conditions and fully characterising the obtained materials, a better understanding of the underpinning mechanisms of CD formation and PL mechanisms will be gained, which in turn will lead to improved materials with high QYs.

#### Non-glucose monosaccharide-based fluorescent carbon dots

In addition to glucose, different monosaccharides and polyols have also been utilised as carbon sources for the synthesis of FCD, although this approach is less common.

**Scheme 12:**
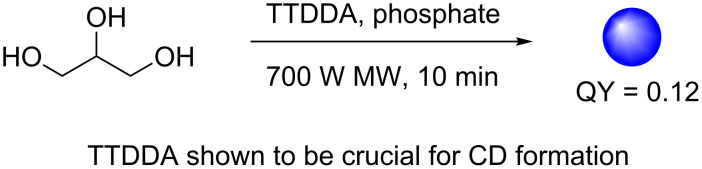
Formation of blue-emissive CDs from the microwave irradiation of glycerol, TTDDA and phosphate.

The ability of glycerol to undergo dehydration and polymerisation in the presence of amino groups makes it a cheap and suitable candidate as a molecular precursor for CD synthesis. To that end, Liu et al. demonstrated in 2011 that the microwave-assisted pyrolysis of glycerol in the presence of TTDDA afforded blue-emissive CDs with a QY of 12% (Scheme 12) [43]. The particles had preeminent multicolour emission, which was excitation-dependent. Importantly, the team demonstrated that TTDDA was crucial as a passivating agent for optimal levels of fluorescence. The method was also applicable to other carbon sources such as glucose, sucrose, glucan and starch. The novel nanomaterials were found to be useful in live cell bioimaging applications. The team carried out cell viability studies (MTT assay) and after treatment of HepG2 cells with these multicolour emissive CDs, 100% cell viability was recorded with concentrations of up to 240 μg/mL of the CDs, while significant toxicity was seen at concentrations at and above 400 μg/mL. CDs (100 μg/mL) were also incubated with HepG2 for 24 h and laser scanner confocal microscopy (LCSM) was used to image the internalization of the CDs within the cells using the green, yellow and red channels, demonstrating their utility.

**Scheme 13:**
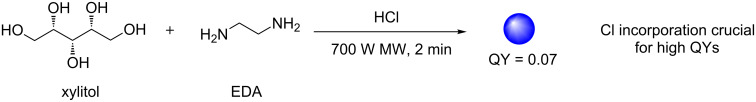
Xylitol-derived N-doped CDs with excellent photostability demonstrating the importance of Cl incorporation to the fluorescence properties.

In a similar fashion, xylitol was used as a CD molecular precursor, in the presence of HCl and ethylene diamine (EDA), in a 2 min microwave-mediated synthesis of CDs developed by Kim et al. [44]. The team successfully demonstrated that to improve the blue emission of the nanoparticles, HCl was crucial. In the absence of HCl as an additive, the QY was only 0.38%, whereas in the presence of HCl, a significant increase of the QY to 7% was observed (Scheme 13). Interestingly, Cl atoms are incorporated as part of the CD structure in as much as 9.14%, based on the elemental analysis, demonstrating that in addition to N-dopant agents, Cl sources such as HCl, are key as SPAs that can improve the PL properties of FCDs. The Cl/N-doped CDs were incubated with WI38 and HeLa cell lines and the cell viability was studied by an MTT assay. It was found that no cytotoxicity was observed up to CD concentrations of 100 μg/mL, while slight levels of toxicity were detected at concentrations of up to 1000 μg/mL. Fluorescence microscopy analysis of HeLa cells treated with CDs at 100 μg/mL for 24 h showed cell internalisation as monitored by their multicolour emissive properties, in addition LCSM confirmed their remarkable photostability too, as long exposure times lead to no obvious photobleaching.

**Scheme 14:**
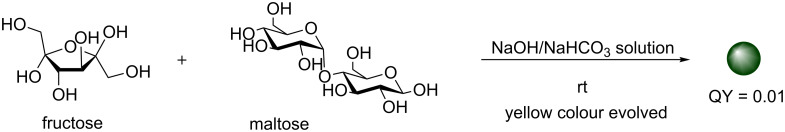
Base-mediated synthesis of CDs with nanocrystalline cores, from fructose and maltose, without forcing reaction conditions.

Fructose and maltose combinations have also been used as an alternative to glucose as the carbon source. The Ostrikov team developed a room temperature preparation of weakly emissive CDs (QY 2%) by mixing a 500 mM aqueous solution of fructose and maltose (a glucose 1,4-linked disaccharide) with a 500 mM solution of NaOH and NaHCO_3_ also dissolved in water (Scheme 14) [45]. The resulting clear mixture was monitored until a colour change towards a yellow colouration was observed after approximately 60 minutes of mixing. Upon excitation by 405 nm lasers, a green fluorescence was recorded. It was found that the concentration of the solution was essential for the formation of CDs. Although this method does not produce highly fluorescent CDs, the example shows that green-emitting CDs can be made in a reaction without either strong heating, N-doping or surface passivation occurring. Also remarkably, the CDs produced by this method were found by HRTEM to have graphite crystallinity. This feature is interesting as, until now, it was thought that this type of crystallinity in a bottom-up constructed nanomaterial was only possible under energy intensive/forcing conditions.

We have already established that an effective method for modulating the properties of CDs is to introduce heteroatoms, with the use of N-dopant agents being the most common. The majority of methods discussed thus far, utilise cheap, readily available neutral carbohydrate such as glucose as the carbon source in combination with a nitrogen-containing molecule. Glucosamine hydrochloride, which is a byproduct from the hydrolysis of chitosan and chitin polysaccharides found on crustacean shells, bears an amine functionality at C-2 and offers all the advantages of glucose, while already containing an N atom. A few examples in the literature have already utilised this sugar as the starting material in the synthesis of CDs with interesting results. One of the earliest examples of the hydrothermal preparation of FCDs using glucosamine hydrochloride was shown by Wang et al. [34]. A one-step process whereby an aqueous (deionised) solution of the amine-containing glucoside was heated in an autoclave to 140 °C for 12 h, which after several days of dialysis, led to strongly green-emitting CDs with a 35 nm average diameter. Interestingly, the authors observed that under the same reaction conditions, glucose did not generate CDs. The authors proposed that polymerisation of glucosamine molecules followed by aromatisation via intramolecular dehydration, leads to a burst of nucleation when the aromatic cluster supersaturation is reached. This burst of nucleation takes place and the carbon nuclei grow to partially nanocrystalline CDs with certain hydrophilic functional groups in the surface. Raman, FTIR and XPS data confirmed the presence of aromatic amines, hydroxy and carboxy groups on the CD surface.

**Scheme 15:**
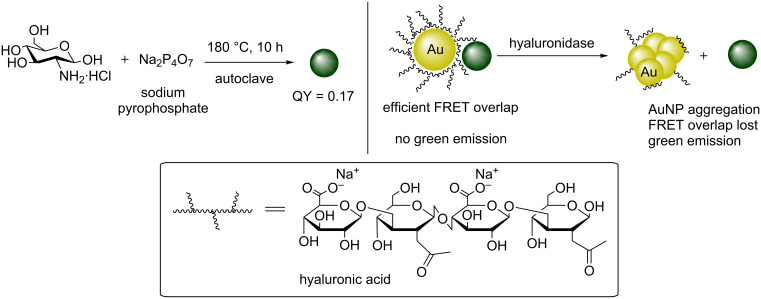
N/P-doped green-emissive CDs working in tandem with hyaluronic acid-coated AuNPs to monitor hyaluronidase activity.

Subsequently, Liu et al. reported the hydrothermal synthesis of amino-functionalised green fluorescent CDs using glucosamine hydrochloride in the presence of excess sodium pyrophosphate (Na_4_P_2_O_7_) (Scheme 15) [19]. The team showed that heating an aqueous mixture of the sugar and Na_4_P_2_O_7_ for 10 h to 180 °C in a Teflon-lined autoclave resulted in green fluorescent N/P-doped CDs with QYs of up to 17% with an excitation independent emission which could be modulated by varying the concentration of Na_4_P_2_O_7_ in the starting mixture. Also, the higher the concentration of pyrophosphate, the less aggregation product was observed. The resultant CDs were then effectively coupled to hyaluronate (a long-chain polymer containing repeating disaccharide units of glucuronate-β1->3-*N*-acetylglucosamine) stabilised gold nanoparticles (AuNPs) and used as a sensitive and selective probe to monitor hyaluronidase enzymatic activity, which is an enzyme that breaks down hyaluronate (Scheme 15). As hyaluronate is used to stabilise the AuNPs, any enzymatic activity that degrades the polymer would result in AuNP aggregation, which in turn modulates the absorption properties of the AuNPs. The latter has a favourable overlap with the emission spectra of CDs, when stabilised. Hence a turn-on of the CD fluorescence is indicative of enzyme activity.

**Scheme 16:**
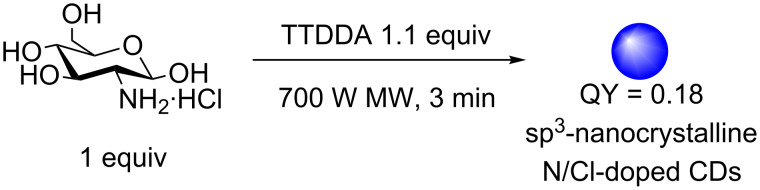
Three-minute microwave synthesis of Cl/N-doped CDs from glucosamine hydrochloride and TTDDA to afford bottom-up synthesised CDs with an sp^3^ nanocrystalline core.

More recently, Galan et al. reported the 3 min one-step synthesis of blue-emitting CDs from glucosamine hydrochloride in the presence of TTDDA using microwave irradiation with QYs of up to 17% (Scheme 16) [46]. While most reported syntheses afford CDs with sp^2^ crystalline or amorphous cores, the team showed that the resultant nanoparticles had an sp^3^ nanocrystalline core, as determined by HRTEM and Raman spectroscopy. The authors attributed this observation to the relatively mild conditions used. They also showed that the presence of HCl was critical for the PL properties of the CD and that the formation of C–Cl bonds, as determined by Raman and FTIR spectroscopy, yielded the chlorine as a crucial auxochrome, which is in agreement to results previously reported by Kim et al. [44].

**Scheme 17:**
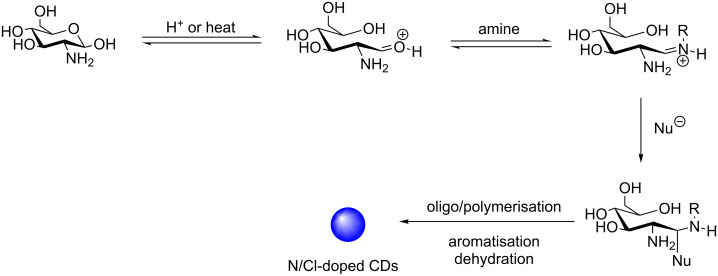
Mechanism for the formation of N/Cl-doped CDs via key aldehyde and iminium intermediates, monitored by ^1^H and ^13^C NMR, FTIR and React-IR studies.

Mechanism studies of the reaction by ^1^H, ^13^C, FTIR and React-IR helped to identify the key reaction intermediates (Scheme 17). The loss of the anomeric proton/carbon with formation of an aldehyde was observed within the first 90 seconds of the reaction, after which time, amide formation and sp^2^-centre formation/aromatisation were also observed. React-IR studies under hydrothermal conditions, but at a lower temperature of 70 °C, helped the team to identify a reactive iminium species, which is formed from the reaction between the sugar aldehyde and an amine present in the reaction mixture, and is a key intermediate in the initial stages of nanoparticle formation. Trapping of the iminium electrophile could allow oligomer formation and dehydration, leading to the formation of the sp^3^-enriched nanocrystalline core. In the second phase of the reaction, following the loss of bulk water, further carbonisation occurs and aromaticity is then generated on the outer layers of the core. Surface passivation by TTDDA can now take place via either incorporation of TTDDA into the surface heteroaromatics or amide bond formation. Amide formation can occur either through surface-bound carboxylic acids reacting directly with an amine (e.g., TTDDA or sugar-derived amine) or through the nucleophilic attack of an alcohol to the iminium electrophile, followed by rearrangement of the resulting imidate.

**Scheme 18:**
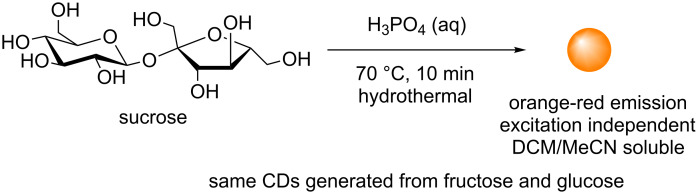
Phosphoric acid-mediated synthesis of orange-red emissive CDs from sucrose.

**Scheme 19:**
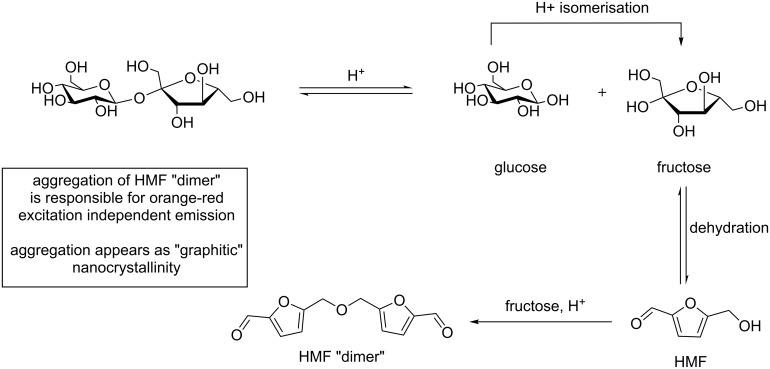
Proposed HMF dimer, and its formation mechanism, that upon aggregations bestows orange-red emissive on sucrose-derived CDs.

The work by Mandal et al. has also recently sought to provide some insights into nanoparticle formation and PL mechanism for sugar-derived CDs [47]. The team studied the reaction between sucrose and H_3_PO_4_ to afford excitation-independent orange-red emissive CDs (Ex = 365 nm), which were readily soluble in organic solvents such as DCM and MeCN (Scheme 18). Mechanistic investigations showed evidence of hydroxymethylfurfural (HMF) derivatives as the major component in the preparation of this type of CD, as evidenced by ^1^H, ^13^C NMR, FTIR and MALDI–MS (Scheme 19).

The authors proposed that initial acid catalysed degradation of the sucrose disaccharide to its monosaccharide constituents fructose and glucose, followed by glucose isomerisation to fructose, leads to HMF formation following three dehydration steps. Indeed, HMF formation has been identified as a dehydration product in reactions with glucose, fructose and sucrose under acidic conditions [48]. Furthermore, the team was able to show that instead of polymeric furfural structures, HMF dimers are produced via the acid-catalysed ether formation between HMF and fructose followed by subsequent dehydration, and undergo aggregation to form fluorescent CDs. Although, it is clear that small changes in the reaction conditions and reagents do have a significant effect in the final nanoparticle properties. These results provide evidence that aggregation of furfural intermediates or other heteroaromatic species could be responsible for the PL and physicochemical properties observed.

### Fluorescent carbon dots synthesised from polysaccharides

Polysaccharides are essentially polymeric sugar molecules composed of monosaccharide units coupled together via glycosidic linkages to form long linear or branched chains. Some of the most common polysaccharides found in nature include cellulose, starch, glycogen or chitin [49]. Upon hydrolysis, these structures break down into smaller fragments such as oligosaccharides or monosaccharide units. Thus, it is unsurprising that these naturally occurring materials have also been used as CD precursors. Many CD syntheses report the use of biomass, particularly sourced from plant matter, which is essentially a huge source of naturally derived polysaccharides combined with smaller amounts of other organic molecules, e.g., amino acids, which can act as dopant agents. Some examples include the use of garlic [50], orange juice [51], onion waste [52] and general kitchen waste [53]. For the purpose of this review, we will concentrate on describing examples where defined and commercially available polysaccharides are used for the synthesis of FCDs and how these materials compare to CDs made using their monomeric counterparts.

**Scheme 20:**
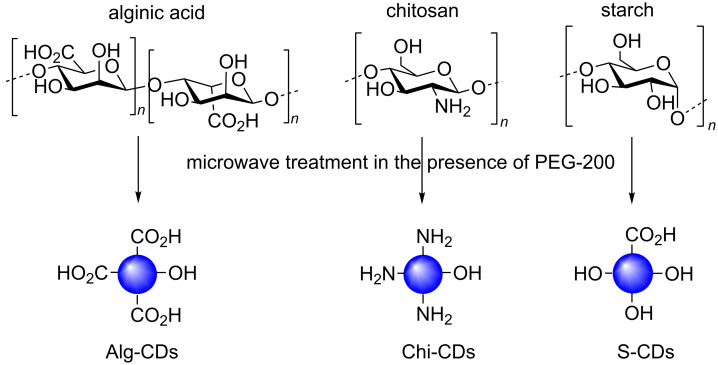
Different polysaccharide-derived CDs in the presence of PEG-200 and how the starting material composition is conferred to the CD products.

Many different polysaccharides with different elemental composition and structural morphologies are available and as seen for monosaccharide-derived CDs, the different features and functional groups present in those distinct carbohydrate chains, will have an effect in the final properties of the CDs synthesised from them. Pramanik et al. exploited this hypothesis in the synthesis of CDs from three different polysaccharides: chitosan (Chi-CDs), alginic acid (Alg-CDs) and starch (S-CDs) in the presence of PEG-200 under identical microwave conditions (Scheme 20) [54]. TEM analysis of the samples highlighted that a range of morphologies and sizes were obtained depending on the polymer used. For example, S-CDs afforded the smallest particle size distribution (1–2 nm) but little morphological uniformity. On the other hand, Chi-CDs appear to have a distinctly spherical morphology with a size range of 2–10 nm and Alg-CDs also exhibit a distinct spherical morphology with a size range of 2–4 nm. Interestingly, an inverse correlation between the size of the CD and the fluorescence output was established, the smallest S-CDs gave the best fluorescence intensity of the three samples, while the largest Chi-CDs had the lowest. FTIR analysis provided evidence that the starting polysaccharide functional group composition is conferred onto the CDs. For instance, alginic acid has one carboxylic acid group per monomer unit, whereas chitosan is an amine-containing polysaccharide; analysis of the different CDs showed higher intensities for peaks attributed to carboxylic acid C=O bonds in both Alg-CDs and Chi-CDs. Similarly Chi-CDs showed an abundance of amine functionality, while S-CDs spectra had many more signals that could be assigned to alcohol groups and some carboxylic acid functionality, with the latter probably generated during the reaction. The authors further demonstrated the applicability of the different materials in heavy metal sensing. To that end, each CD sample was exposed to the same concentration (0.001 M) of divalent metal cations Cu^2+^, Cd^2+^, Sn^2+^ or Zn^2+^ in solution and the fluorescence response monitored. The starch-based S-CDs showed an interesting PL response, whereas the fluorescence output increased when in solution with all metal ions tested, in the case of Cu^2+^, a significant reduction in fluorescence was recorded. The authors proposed that Cu^2+^, due to its paramagnetic nature, could quench the S-CD fluorescence via a photoinduced electron transfer mechanism, in which Cu^2+^ is reduced to Cu^+^. The presence of Cu^+^ was confirmed by selected area electron diffraction (SAED) in the TEM analysis of the CD surface.

**Scheme 21:**
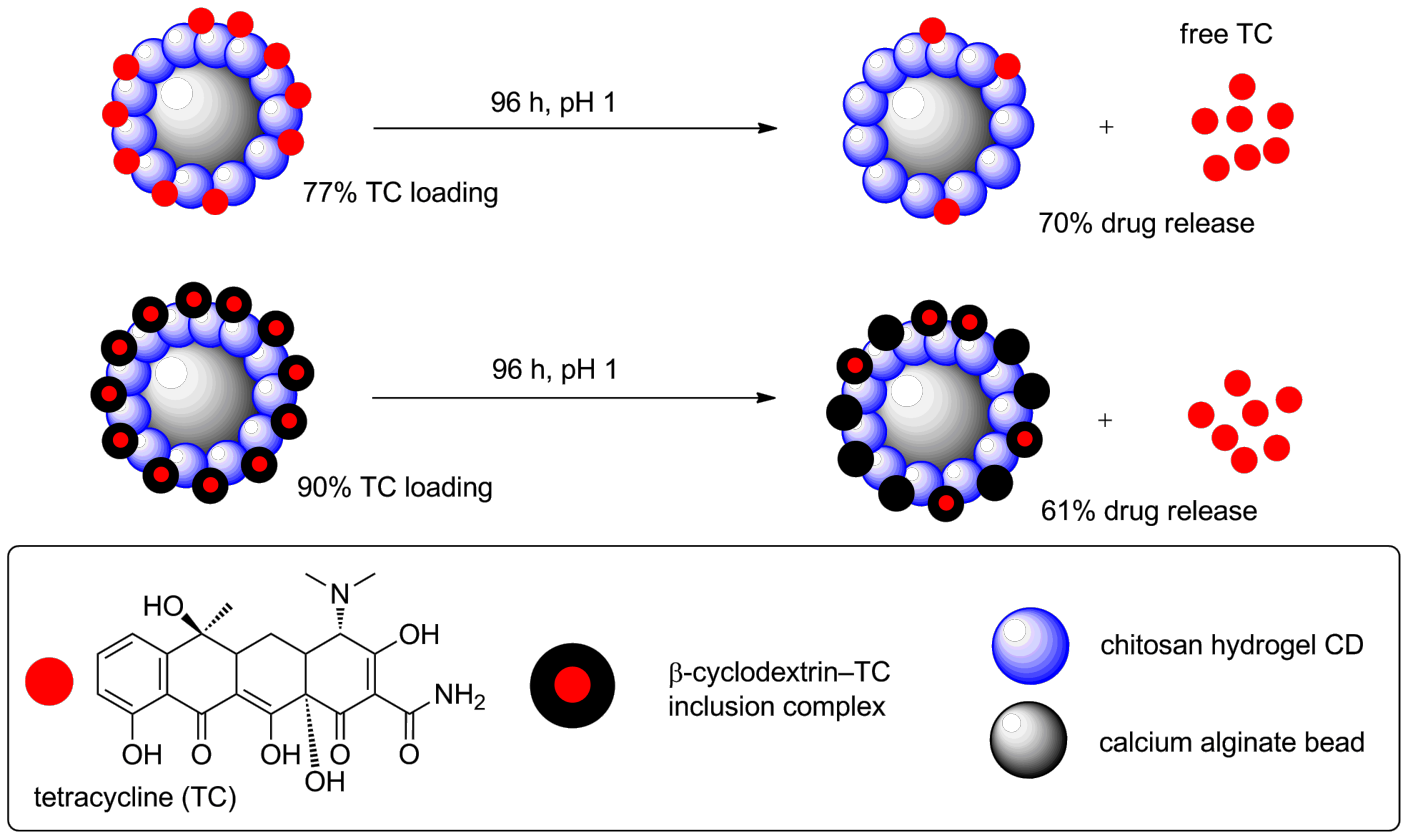
Tetracycline release profiles for differentially-decorated CDs.

CDs from chitosan hydrogels have also been reported by Chowdhury et al. [55]. The hydrogels were synthesised from a mixture of acetic acid, glycerol and chitosan, as a more stable starting material for CDs. Microwave irradiation of the hydrogel yielded UV-blue emissive CDs with a range of sizes from 0.6–8.7 nm (as determined by DLS). Zeta-potential analysis yielded a value of +27 mV, indicative of an abundance of amino groups and as expected from an amino group containing chitosan starting material. In addition, the group also investigated CDs prepared from chitosan/Ag and chitosan/Au nanocomposites, which were incorporated while preparing the chitosan hydrogels. It was observed that although the emission of the new CDs was broad and less well defined, there was an enhancement in the PL emission for the Ag or Au-doped CDs. Subsequently in 2014, the same group was able to show that coating of calcium alginate (CA) beads with chitosan hydrogel-based CDs yielded a new nanomaterial that could be employed as a pH-responsive drug-delivery vehicle (Scheme 21) [56]. The CDs were used as a protective layer onto the CA beads and tetracycline (TC) was loaded onto the CD–CA beads. It was shown that a two-fold increase on drug loading was seen when compared to uncoated beads. Subsequently, TC release at a range of pH values was studied over a 96 h period and it was found that 70% of TC release takes place at low pH (pH 1) when compared to 36% release at pH 7 and 27% at pH 12. In order to improve the drug delivery profile of the complex, the authors developed a β-cyclodextrin/tetracycline (β-TC) host–guest inclusion complex, which allows a second “barrier” of release. The pre-formulated β-TC complex was loaded onto the CD–CA beads and not only were higher loading levels measured (90%), but also a slower rate of TC release at each pH value was recorded, as expected from a more stable drug/nanocomplex. The results reported here are good examples of the potential applications of amine-coated CDs as important components in drug-delivery applications.

Chitin, which is a cheap and readily available linear polysaccharide comprised of β-1,4-linked *N*-acetyl-D-glucosamine units, is the second most abundant biopolymer in nature and forms the backbone of crustaceans and insects exoskeleton and is also found in the cell wall of yeast and fungi [57]. Chitin is also the precursor of chitosan, which is formed by N-deacetylation to partially free amino groups, and is notoriously insoluble in water. Despite this fact, Shchipunov et al. demonstrated in 2015, the first hydrothermal synthesis of CDs derived from chitin in a Teflon-lined autoclave at 180 °C for 3 h in the presence of HNO_3_, all in deionised water [58]. The CDs produced in this manner were purified from unreacted/unsolubilised chitin via several filtration, centrifugation and dialysis steps. The N-doped CDs were blue-emitting under UV excitation with apparent long-term, bench stable fluorescence. These results might suggest further opportunities in the field for these type of less water soluble N-containing polysaccharides.

**Scheme 22:**
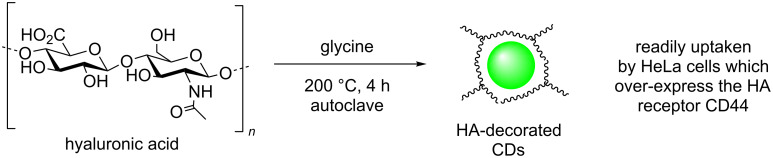
Hyaluronic acid (HA) and glycine-derived CDs, suspected to be decorated in unreacted HA, allowing receptor-mediated cell uptake.

Hyaluronic acid is another N-containing polysaccharide composed of repeating dimeric units of glucoronic acid and *N*-acetyl-D-glucosamine units and which forms the core of complex proteoglycan aggregates found in the extracellular matrix [57]. The team of Du and Shao et al. reported the synthesis of N-doped hyaluronic acid-derived CDs and their application as drug delivery vectors [59]. Following standard hydrothermal synthetic procedures as previously described for other CD preparations, hyaluronate was heated in a Teflon-lined autoclave in the presence of glycine, which was found to be key, to 200 °C for 4 h to yield CDs of under 10 nm in size (Scheme 22). Structural analysis of the resultant CDs indicated the presence of carbonyl-containing functional groups such as carboxylic acids and amides, which coated a graphitic-type core. The nanoparticles exhibit excitation-dependent emission and were blue-emissive under UV excitation, but green when excited at 496 nm. Although no NMR characterisation was carried out on the samples, the authors proposed that due to the polymeric nature of the starting material, the resulting N-doped CD cores might be decorated by unreacted/fragmented hyaluronic acid (HA–CD). Subsequent cell feeding experiments with HA–CD with HeLa and U251 cells, revealed that upon internalisation the CDs where found to localise in the cytoplasm and particularly around the nucleus. Due to the large amounts of internalisation a receptor-mediated endocytosis was proposed. The particles were used as fluorescent probes to target CD44 high expression in tumour cells, opening the door for these types of polysaccharide-based nanomaterials in other targeted live cell labelling, imaging and drug-delivery applications.

**Scheme 23:**
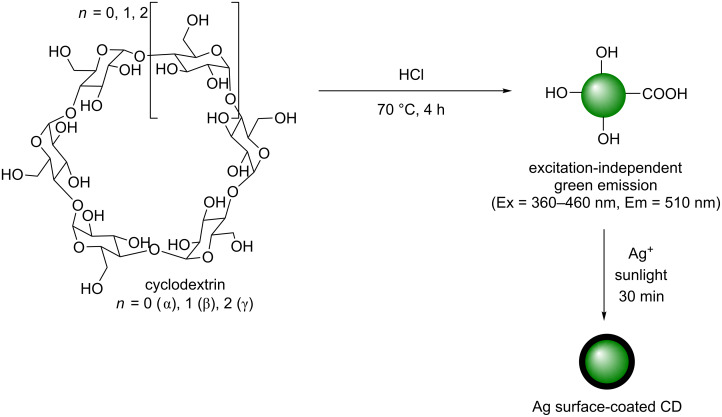
Cyclodextrin-derived CDs used for detection of Ag^+^ ions in solution, based on the formal reduction of Ag^+^ to afford plasmonic Ag.

In addition to amine containing polysaccharide, other neutral carbohydrate-based polymers have also been reported in the synthesis of CDs. Cyclodextrin is a cyclic glucose polymer that is commonly available in its α, β and γ forms, each corresponding to the number of glucose units (6, 7 and 8, respectively). In 2014, Wang et al. reported the synthesis of CDs from each of the different cyclodextrins via an acidic, hydrothermal treatment at 70 °C for 4 h (Scheme 23) [60]. Reaction of each type of cyclodextrin afforded quasi-spherical nanoparticles with a size range of 2.5 ± 0.8 nm and with an amorphous carbon core. The materials obtained had a range of alcohol and carbonyl-containing functionalities present on their surface. QYs were measured to range from 9% to 13%, which were dependant on the type of cyclodextrin utilised, with each CD showing a green emission under UV irradiation and excitation-independent emission from 360 to 460 nm excitation, which is typical of a uniform morphology. The authors proposed that the uniform emission could be attributed to either the uniform size distribution or the uniform surface state giving a single quantum dot-like emission profile. This interesting report highlights the fact that a reducing sugar is not essential to produce CDs and that under acidic and forcing conditions this type of starting materials can still undergo acetal hydrolysis, dehydration, aromatisation and carbonisation to yield CDs. The resulting cyclodextrin-derived CDs were then used for the detection of Ag^+^ ions in solution. It was found initially that mixing AgNO_3_ in an aqueous solution of CDs in sunlight resulted in the formal reduction of Ag^+^ to elemental Ag^0^, which was thought to proceed via the adhesion of Ag^+^ to the CD surface, followed by reduction in the presence of sunlight, which promotes the excitation of the reducing electron to a higher energetic state (Scheme 23). Through UV–vis absorbance and TEM measurements it was evident that a surface layer of plasmonic Ag existed on their surface. The PL intensity of the CDs was modified in a linear manner with Ag^+^ concentrations, and as such the nanoparticles could be utilised as a fluorescence probe to detect Ag^+^ in solution up to concentrations of 0–25 μM.

**Scheme 24:**
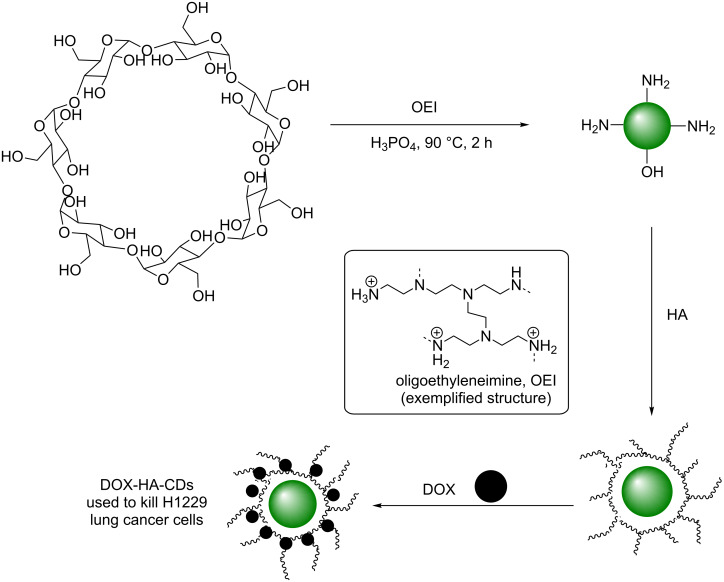
Cyclodextrin and OEI-derived CDs, coated with hyaluronic acid and DOX, to produce an effective lung cancer cell drug-delivery vehicle.

β-Cyclodextrin has also been utilised in the synthesis of CDs using a surface passivation and inorganic dehydration method. The groups of Yang and Teo et al. demonstrated that the synthesis of excitation-independent green emissive CDs could be achieved through the reaction of β-cyclodextrin in the presence of oligoethyleneimine (OEI) and phosphoric acid under thermolysis conditions (90 °C) for 2 h (Scheme 24) [61]. It was demonstrated that the presence of phosphoric acid was crucial for the formation of fluorescent CDs, as the control reaction, in the absence of acid, did not produce emissive CDs. AFM and TEM indicated quasi-spherical CDs of 2–4 nm, while FTIR and XPS indicated that nitro groups were present within the CD structure. The green-emissive CDs were photostable at a wide range of pH values (1–13) and over long-exposure to excitation sources. These results indicated the advantages of inorganic-ion mediated dehydration and the use of N-doping via surface passivation to achieve QYs up to 30%. Due to the use of OEI, the CDs were positively charged as measured by ZP and as a result the novel nanomaterial could form nanocomplexes with negatively-charged polymers. To demonstrate their applicability, CDs were successfully decorated with hyaluronic acid (HA), a negatively-charged polysaccharide, and shown by AFM, DLS and TEM, to have formed nano-aggregates of up to 250 nm. Interestingly, the emissive properties of the CDs were unchanged upon complexation to HA. The resultant nano-aggregates were then loaded with doxorubicin (DOX) and a strong correlation between dose and cell death was demonstrated in lung cancer H1299 cells (Scheme 24).

**Scheme 25:**
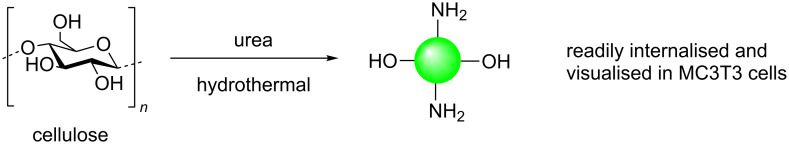
Cellulose and urea-derived N-doped CDs with green-emissive fluorescence.

Cellulose is the most abundant organic molecule on Earth and is a linear polysaccharide comprised of repeating β-1,4-linked glucose units. Similarly to cyclodextrin, cellulose does not contain N-functionalities, which has been shown to be crucial for superior PL properties in CDs. In order to exploit the abundance, renewable and cheap advantages offered by using cellulose as the starting material, an N-doping strategy is needed. The group of Yao described recently the formation of CDs from cellulose (the specific type is not defined in the original report) via hydrothermal treatment in the presence of urea (Scheme 25) [62]. The resultant CDs were blue-green emissive with excitation-dependent emission and a QY of up to 21%. The high QY and favourable fluorescence properties were, in-part, attributed to the presence of auxochromic N within the architecture of the CDs. Subsequent CD internalisation experiments in MC3T3 osteoblast cells indicated that after exposure times of up to 24 h, the cell viability was unchanged when using concentrations of CDs of up to 250 µg/mL. LCSM experiments showed that the CDs were readily internalised into the cells and could potentially find uses in drug-delivery applications.

## Conclusion

FCDs have only been around for a little over a decade and yet, it has become clear that these novel fluorescent nanomaterials have tremendous potential in many applications such as metal sensing, photocatalysis and as probes for bioimaging and biomedical applications and they offer a cheaper and non-toxic alternative to other metal-based fluorescent nanomaterials, e.g., semiconductor QDs. In this review, we have described a number of synthetic approaches to access FCDs using mono-, oligo- and polysaccharides, as cheap and readily available starting materials and the data has been collated in Table 1. Methods described include thermal decomposition, chemical or hydrothermal oxidation under autoclave, ultrasonic or microwave-assisted conditions. The presence of defects in the CD structure has been proposed to be important with regards to their PL properties. Additionally, the use of surface passivating agents to provide uniform PL trapping sites on the CD surface and the introduction of electron-donating heteroatoms as dopant agents, have been shown to improve and help tune the PL properties of these interesting nanomaterials. Not one synthesis is the same, it has been made evident that small changes in the synthetic scheme employed to access CDs, have an impact on the final chemical and physical properties of the nanoparticles obtained (See Table 1). Thus, careful consideration needs to be given to the type of carbon source used (carbohydrates being inherently heterogeneous provide an abundant and cheap source to be explored among other materials), reagent ratios/concentration, presence or absence of dopant agent/s (N, P, S or B) and their sources, and type of chemical process employed. Although a full mechanism of CD formation has not been elucidated to date, initial mechanistic studies on the formation of CDs from carbohydrates, have suggested that carbohydrate ring-opening to the aldehyde, which can then react with available nucleophiles in the reaction mixture is key. Subsequent dehydration/aromatization events can take place, which lead to the production of N-heteroaromatic structures on the CD surface. The ability to tune the CD synthesis to produce different nanodots, offers unique opportunities and renders these materials amenable to a wide range of applications, as we have briefly described in this review. On the other hand, CD quantum yields are still lower in comparison to their direct competitors (semiconductor QDs) and efforts are currently being devoted to improve their PL properties. As we gain a better understanding at the molecular level of the mechanism of photoluminescence and chemical formation of these exciting nanomaterials, we will be able to devise procedures to access designer materials for specific applications. It is clear that the future of this field is “CD” bright.

**Table 1:** Summary of carbohydrate-derived CDs synthetic protocols and properties.

Carbohydrate	Heteroatom dopant/SPA^a^	Synthetic conditions	Fluorescence profile^b^	Size [nm]	Crystallinity	Principle functionality	Ref.

glucose	PEG-200	microwave	blue to green (Dep)	2–4	amorphous	C=C, C=O (acid), OH, C-O	[18]
glucosamine HCl	Na_4_P_2_O_7_	Teflon-autoclave, reflux	green (Ind)	4	–	C=C, C=O, C-O, C-N, -OH, -NH_2_	[19]
not specified	various	hydrothermal	blue to Red	various	various	various	[20]
glucose	TTDDA	H_2_SO_4_, HNO_3_ reflux	blue (Dep)	5	graphitic	C=C, C=O (acid/amide), OH, C-O	[23]
glucose	KH_2_PO_4_	Teflon-autoclave, reflux	blue or green (Dep)	2–5	graphitic	C=C, C=O (acid), OH, C-O	[29]
glucose	phosphate	microwave	blue to green (Dep)	2	–	C=C, C=O (acid), OH, C-O	[31]
glucose	NH_3_	ultrasonic	blue (Dep)	10	graphitic	C=C, C=O (acid), OH, N-aromatics, C-O	[32]
glucose	tryptophan	microwave	blue (Ind)	20	–	C=C, C=O (acid), OH, N-aromatics, C-O	[33]
glucosamine HCl	–	Teflon-autoclave, reflux	green (Ind)	30	crystalline	C=C, C=O, C-N, C-O, O-H	[34]
glucose	PEG-diamine	H^+^/ultrasonic	blue (-)	10	–	C=C, C=O (acid/amide), OH, C-O, N-H	[36]
glucose	boric acid	Teflon-autoclave, reflux	blue (Dep)	3–5	–	C=C, C=O (acid), -OH, B-OH	[38]
glucose	glutathione	hydrothermal	blue to green (Dep)	2.5	amorphous	C=C, C=O, C-O, N-H, Oxidised S	[39]
glucose	EDA, conc. H_3_PO_4_	hydrothermal	blue to green (Dep)	10	hollow	C=C, C=O, C=N, -OH, P=O, P-C	[40]
glucose	NH_3_, H_3_PO_4_	Teflon-autoclave, reflux	blue (Dep)	3	graphitic	C=C, C=O, P-C, P-N, P-O	[41]
glucose	TTDDA or dopamine	hydrothermal	blue or green (Dep)	2–7	crystalline	C=C, C=O, -OH, -NH_2_	[42]
glycerol	TTDDA	microwave	blue to green (Dep)	3.5	amorphous	C=C, C=O (amide), -OH, -NH_2_	[43]
xylitol	EDA, HCl	microwave	blue (Dep)	4–5	graphitic	C=C, C=O (amide), -OH, C-N, -Cl	[44]
fructose/maltose	–	NaOH/NaHCO_3_, rt	green (Dep)	3–5	graphitic	C=C, C=O, C-O, -OH	[45]
glucosamine HCl	TTDDA	microwave	blue (Dep)	2–5	sp^3^ crystalline	C=C, C=O (amide), C-O, C-N, C-Cl	[46]
sucrose	–	H_3_PO_4,_ hydrothermal	orange-red (Ind) associated to HMF dimer aggregation	4	graphitic (molecular crystallinity)	C=C, C=O (acid), C-O, -OH	[47]
several polysaccharides	PEG-200	microwave	blue (Dep)	1–10 (substrate dependent)	–	substrate dependent	[54]
chitosan	glycerol, AcOH hydrogel	microwave	UV to blue (Dep)	1–8	–	C=C, -NH_2_, C-O, -OH	[55–56]
chitin	–	HNO_3_, Teflon-autoclave, reflux	blue (Dep)	4-8	graphitic	C=C, C=O (amide), -NH_2_, -OH	[58]
hyaluronic acid	glycine	Teflon-autoclave, reflux	blue (Dep)	2–4	graphitic	C=C, C=O (amide), -NH_2_, C-O	[59]
cyclodextrin	–	HCl, hydrothermal	green (Ind)	2.5	amorphous	C=C, C=O (acid), C-O, -OH	[60]
cyclodextrin	OEI	hydrothermal	green (Ind)	2–4	–	C=C, C=O (anhydride, amide), C-O, -OH, -NH_2_	[61]
cellulose	urea	hydrothermal	blue (Dep)	4	graphitic	C=C, C=O (amide), C-N, C-O, -NH_2_, -OH	[62]

^a^Surface passivating agent, ^b^major fluorescence emission range highlighted; Ind = excitation-independent emission, Dep = excitation-dependent emission.
