# Correction: Fluorescent carbon dots from mono- and polysaccharides: synthesis, properties and applications

**DOI:** 10.3762/bjoc.13.112

**Published:** 2017-06-13

**Authors:** Stephen Hill, M Carmen Galan

**Affiliations:** 1 School of Chemistry, University of Bristol, Cantock’s Close, Bristol BS8 1TS, UK

**Keywords:** fluorescent carbon dots, monosaccharides, nanomaterials, nanotechnology applications, polysaccharides

Our original publication showns some errors in the structures in Schemes 9, 15, 20, and 22. The corrected schemes are shown in this Correction.

The wrong configuration was depicted for C-4 (carrying the OH group) in the pyranose ring of doxorubicin in Scheme 9; the corrected scheme (Scheme 1) is shown below:

**Scheme 1:**
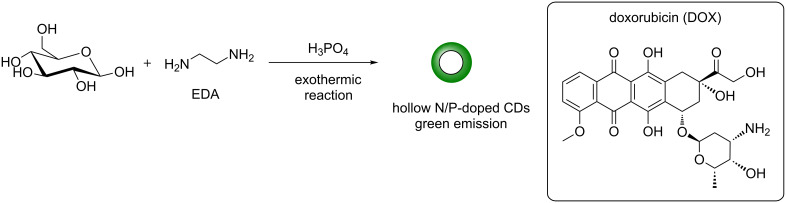
Corrected Scheme 9 of the original article. N/P-doped hollow CDs for efficient drug delivery of doxorubicin.

The NH group was missing at C-2 of the GlcNAc residues in Scheme 15; the corrected scheme (Scheme 2) is shown below:

**Scheme 2:**
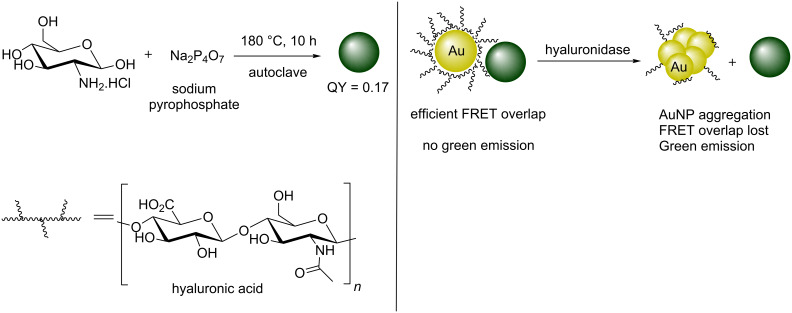
Corrected Scheme 15 of the original article. N/P-doped green-emissive CDs working in tandem with hyaluronic acid-coated AuNPs to monitor hyaluronidase activity.

The carbohydrate polymers in Schemes 20 and 22 were depicted as poly-peroxide with one oxygen atom too many in the repeating unit, repectively; the corrected schemes (Scheme 3 and Scheme 4) are shown below:

**Scheme 3:**
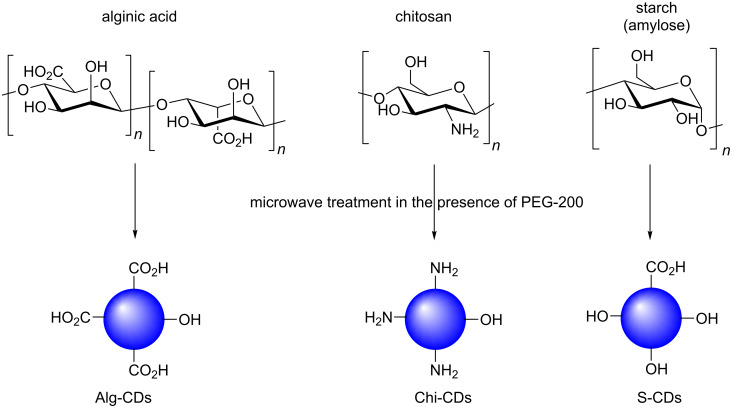
Corrected Scheme 20 of the original article. Different polysaccharide-derived CDs in the presence of PEG-200 and how the starting material composition is conferred to the CD products.

**Scheme 4:**
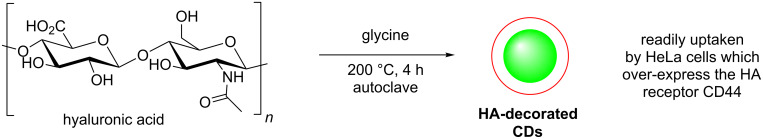
Corrected Scheme 22 of the original article. Hyaluronic acid (HA) and glycine-derived CDs, suspected to be decorated in unreacted HA, allowing receptor-mediated cell uptake.

